# CHANGES IN CARE TRAJECTORIES DURING THE COVID-19 PANDEMIC AMONG OLDER ADULTS WITH CARE NEEDS

**DOI:** 10.1093/geroni/igae098.2326

**Published:** 2024-12-31

**Authors:** Amanda Leggett, Yulya Truskinovsky, Vicki Freedman, Emily Wiemers, Jennifer Corman, Geoffrey Hoffman

**Affiliations:** Wayne State University, Detroit, Michigan, United States; Wayne State University, Detroit, Michigan, United States; University of Michigan, Ann Arbor, Michigan, United States; Syracuse University, Syracuse, New York, United States; Jennifer C. Corman Consulting, Colombus, Ohio, United States; University of Michigan, Ann Arbor, Michigan, United States

## Abstract

**Background and Objectives:**

This study examined whether the progression of care after the onset of an activity limitation changed during the COVID-19 pandemic. Because the pandemic caused widespread disruptions in care, we considered changes in care type and intensity.

**Research Design and Methods:**

We used longitudinal data from the National Health and Aging Trends Study between 2015-2022 to examine trajectories of care after the onset of an activity limitation defined as having difficulty or getting help with any self-care or mobility activity or having probable dementia [N=4,191]. Outcomes included use of paid and unpaid help, number of paid and unpaid helpers, hours of paid and unpaid help, living in a residential care setting, living with adult children, and receiving no care. We estimated trajectories of care using mixed effects models and models with individual fixed effects and compared trajectories before and after the onset of the pandemic.

**Results:**

We found no changes in whether individuals received any paid or unpaid care but we found a reduction in the number of paid and unpaid caregivers and mixed evidence on changes in hours of paid and unpaid care in the post-pandemic period. We did not find evidence that changes in care trajectories during the pandemic differed by years since the onset of the activity limitation.

**Discussion and Implications:**

Reductions in the number of caregivers without a reduction in hours of care implies more intensive caregiving demands among caregivers during the pandemic with potential implications for the well-being of caregivers and care recipients.

